# Molecular characterization of high-risk human papillomavirus (HR-HPV) in women in Lomé, Togo

**DOI:** 10.1186/s12879-021-05956-5

**Published:** 2021-03-19

**Authors:** Akouélé P. Kuassi-Kpede, Essolakina Dolou, Théodora M. Zohoncon, Ina Marie Angèle Traore, Gnatoulma Katawa, R. Alice Ouedraogo, Esther Mah Traore, Prosper Bado, T. Clarisse Ouedraogo, Florencia Wendkuuni Djigma, Simplice Damintoti Karou, Jacques Simpore

**Affiliations:** 1 Molecular Biology and Genetics Laboratory (LABIOGENE), Department of Biochemistry and Microbiology, University JOSEPH KI-ZERBO, P.O. Box 7021, Ouagadougou 03, Burkina Faso; 2 Pietro Annigoni Biomolecular Research Centre (CERBA), P.O. Box 364, Ouagadougou 01, Burkina Faso; 3 Ecole Supérieure des Techniques Biologiques et Alimentaires (ESTBA-UL), University of Lomé, Lomé, Togo

**Keywords:** HR-HPV, Genotypes, PCR, Lomé, Togo

## Abstract

**Background:**

The causative agent of cervical cancer referred to as Human papillomavirus (HPV) remains a real public health problem. Many countries in West Africa, such as Togo have no data on the high-risk HPV (HR-HPV) infection and genotypes distribution. In order to fill the knowledge gap in the field in Togo, the main objective of this study was to determine the prevalence of pre-cancerous lesions of the cervix and HR-HPV genotypes among Togolese women.

**Methods:**

Samples were collected from 240 women by introducing a swab in the cervix. Then, the screening of precancerous cervical lesions using the visual inspection with acetic acid and lugol (VIA / VIL) was conducted. The HR-HPV genotypes were characterised by real-time multiplex PCR.

**Results:**

Out of 240 women recruited, 128 (53.3%) were infected by HR-HPV. The most common genotypes were HPV 56 (22.7%), followed by HPV 51 (20.3%), HPV 31 (19.5%), HPV 52 (18.8%) and HPV 35 (17.2%). The least common genotypes were HPV 33 (2.3%) and HPV 16 (2.3%). Among the women, 1.3% (3/240) were positive to VIA/VIL.

**Conclusion:**

This study allowed HR-HPV genotypes to be characterised for the first time in Lomé, Togo. This will help in mapping the HR-HPV genotypes in West Africa.

**Supplementary Information:**

The online version contains supplementary material available at 10.1186/s12879-021-05956-5.

## Background

Infection by papillomavirus is considered as a sexually transmitted disease (STD) affecting the general population [1]. According to the World Health Organization (WHO), infections due to HPV affect 660 million people in the world and constitute a major public health problem [2]. Human papillomavirus (HPV) is responsible for cervical cancer and is a virus resistant to cold, organic solvents and detergents, with limited sensitivity to heat or even to chlorine used in pools [1]. Cervical cancer is the first cause of mortality by cancer in women in many developing countries. It is consecutive to a persistent infection due to high risk HPV (HR-HPV) [3]. There is an annual incidence of 500,000 cases and around 250,000 deaths annually [4]. In developed countries, there is a decrease of the mortality rate linked to cervical cancer thanks to systematic screening and treatment of pre-cancerous lesions [5]. Cervical cancer is also the first cause of cancer in women in sub-Saharan Africa. Studies conducted in the world and in Africa on groups of women have shown that the prevalence and genotypes of HPV vary according to the regions of the world. Prophylactic vaccines currently available in Africa and particularly ordered in Togo are Gardasil4 and Cervarix. Cervarix offers protection against HPV types 16 and 18. Gardasil4 protects against four types of HPV, including types 16 and 18 which are responsible for cervical cancer, and types 6 and 11 which cause genital warts. The genotypes targeted by these vaccines are sometimes not the most common in certain African regions. Studies have shown that in some African countries like Burkina-Faso, Benin, Mali, Senegal, and the Côte d’Ivoire, these two genotypes were not the most predominant in women [3, 6, 7]. Under the scope of these observations, the current study has been conducted in Lomé, the capital city of Togo to determine the prevalence of HR-HPV and different genotypes.

## Methods

This transversal study is both descriptive and analytical and was conducted between December 2016 and March 2017 in the maritime region of Lomé, the economic capital city of Togo. This city is located in the south of the country, covering an area of 6100 km^2^.

### Population and sample

The population consisted of all the women who attended the gynaecological service from four hospitals: *Clinique Victoire*, *Centre Médical de Santé Source de Vie*, *Clinique Autel d’Elie* and *Centre de Santé Saint Camille*. From this population, a random sample of 240 sexually active women was taken to collect endocervical swab samples. Each woman responded to a questionnaire (Supplementary file) to provide information on her socioeconomic status, behavioural and sexual habits. All the 240 patients were screened for precancerous cervical lesions using the visual inspection with acetic acid and lugol before molecular detection of High-risk HPV.

This study was approved by the Ethics Committee for Research in Health of Togo (CNE) and Burkina Faso (CERS). All patients were asked to give their written consent before sampling.

### Endocervical samples collection

The gynaecologist placed the patients in the lithotomy position and examined their external genitalia. Then, he inserted a sterile speculum into the vagina and introduced a cotton swab into the endocervical canal. With three counter clockwise movements, he collected cells from the squamocolumnar junction (SCJ). The swab sample was introduced in a sterile Eppendorf tube with a transport medium provided with the DNA-Sorb-A kit (Sacace Biotechnologies, Como, Italy). All swabs samples were then collected and preserved at − 20 °C in one of the 4 health centres before being transported to Ouagadougou (Burkina Faso) at the Pietro Annigoni Biomolecular Research Centre (CERBA) for molecular analysis.

### Screening of cervical precancerous lesions by VIA/VIL

Following cervical swabs samples collection for HR-HPV detection, the screening of cervical precancerous lesions or dysplasia was performed by the gynaecologist using visual inspection of the cervix with acetic acid (VIA) and then with lugol (VIL). VIA was conducted by applying a 4% acetic acid solution to the cervix. The test was considered positive if distinct, well-defined, and dense acetowhite areas were seen or as negative if there were no acetowhite lesions. To conduct VIL, the gynaecologist delicately applied 10% lugol solution to the cervix. Clinically, the test was considered as “negative” for dysplasia when no unstained lesion area was observed, or as “positive” when an unstained lesion area is observed.

### Detection of HR-HPV genotypes by real-time-PCR (RT-PCR)

Genomic DNA was isolated using the DNA-Sorb-A kit (Sacace Biotechnologies, Como, Italy) in compliance with the instruction manual**.**

DNA was amplified by multiplex RT-PCR using the « HPV Genotypes 14 Real-TM Quant » V67–100 FRT kit of SACACE biotechnologies®, Italia following the protocol provided by the manufacturer. The kit allowed us to detect 14 genotypes of HR-HPV: 16, 18, 31, 33, 35, 39, 45, 51, 52, 56, 58, 59, 66 and 68.

Pre-PCR steps consisted in preparing the Mix containing the DNA polymerase, primers, and deoxy ribonucleotide triphosphates. We introduced into each tube, 15 μL of the Mix solution and 10 μL of the DNA extract to obtain a volume of 25 μL. This mixture is placed on a SaCycler-96 Real Time PCR v.7.3 (Sacace Biotechnologies, Italia) platform for amplification.

The amplification schedule was as follows: one (1) cycle at 95 °C for 15 min; five (5) cycles at 95 °C for 5 s, followed by incubation at 60 °C for 20 s and at 72 °C for 15 s; and finally 40 cycles at 95 °C for 5 s followed by 60 °C for 30 s and 72 °C for 15 s.

### Potential factors associated with HPV infection

Based on the answers given by women to the questionnaire, we analysed the relation between some factors and the carrying of HPV infection. These were: age, age at first intercourse, number of sexual partners and the result of VIA/VIL.

### Data analysis

The data were processed and analysed using SPSS software version 21.0, the Microsoft EXCEL 2010 software and Epi Info software version 6. Chi-square was used to compare proportions and averages. The difference was statistically significant for *p* < 0.05.

## Results

### Socio-demographic, sexual, and behavioural characterisation of the women subjects

This study was conducted on 240 endocervical samples from sexually active women. We used the results of the questionnaire to analyse the socio-demographic, sexual, and behavioural characterisation of the women. The age of the women varied from 17 to 67 years with an average of 37.2 ± 12.7 years. In 39.6 and 35% of the cases, the women had secondary and higher education level respectively. The others (6.3%) were illiterate or had a primary education level (19.2%). The unemployed women and housewives represented respectively 49.6, and 10.8%. Only 21.2% women were salaried, 2.1% were retired and 16.3% were students. Married women represented 48.8%. The age at which occurred the first sexual relation varied between 8 and 30 years. More than half of women in this study did not use condoms during sexual intercourse (59.2%). Those having at least one sexual intercourse a week represented 80.76%. Table 1 shows the socio-demographic, sexual and behavioural characteristics of these women.

**Table 1:** Clinical and socio-demographic characteristics of the 240 women

Characteristics	Number	Percentage (%)
**Age (years)**		
**<** 30	89	20.3
30–49	104	47.5
> 49	47	32.2
**Level of education**		
Illiterate	15	6.3
Primary	46	19.2
Secondary	95	39.6
University	84	35.0
**Marital status**		
Single	98	40.8
Divorced	10	4.2
Married	117	48.8
Widow	15	6.3
**Occupation**		
Student	39	16.3
Salaried	51	21.2
Housewives	26	10.8
Retired	5	2.1
Unemployed	119	49.6
**Condom Use**		
Each time	18	7.5
Never	142	59.2
Sometimes	61	25.4
Rarely	19	7.9
**Age of first sexual intercourse** ***n*** **= 238**		
8–18	69	29.0
18–24	155	65.1
24–30	14	5.9
**Frequency of intercourse**		
1 per week	192	80.76
1 per month	48	19.24
**Notion of genital infection**		
Yes	30	12.6
No	210	87.4

### Frequency of precancerous lesions following VIA/VIL

Screening by VIA/VIL conducted on all the 240 women allowed the detection of 1.3% (3/240) dysplastic lesions (Table 2). HPV 56 and 39 were the genotypes found respectively in two VIA/VIL-positive women who were less than 30 years old. The third IVA/IVL-positive woman was a 51-year-old European carrying the HPV 16, 18 and 31 genotypes.

**Table 2:** Results of the screening by VIA/VIL according to HR-HPV infection

VIA/VIL	HPV+		HPV-		
	n	(%)	n	(%)	Total
Positive	3	(1.3)	0	(0.0)	3
Negative	125	(52.1)	112	(46.7)	237
Total	128	(53.3)	112	(46.7)	240

### Frequency of HR-HPV infection and distribution of genotypes

**Fig. 1:**
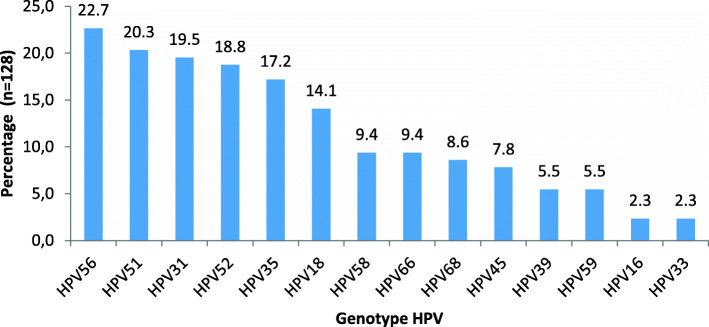
Composition of HR-HPV genotypes among the women

The results of analysis by Multiplex RT-PCR showed that 53.3% (128/240) of women were infected by high-risk HPV. The kit used in the study allowed the characterisation of 14 genotypes of HR-HPV: HPV 16, 18, 31, 33, 35, 39, 45, 51, 52, 56, 58, 59, 66 and 68. The most common genotypes found in women were HPV 56 (22.7%) and HPV 51 (20.3%), followed by HPV 31 (19.5%), HPV 52 (18.8%) and HPV 35 (17.2%). Genotypes 33 and 16 with the same prevalence at 2.3% were the less common. HPV 16 and 18, the target of the vaccines currently available represented together 16.4% of cases. Figure 1 shows the distribution of these genotypes.

The number of HR-HPV genotypes per women ranged from 1 to 5. Considering multiple infections, we observed a total of 204 genotypes in 128 women infected by high-risk HPV. Among the 128 women infected with HR-HPV, 56.0% (72/128) were infected by only one type of HPV. There were 38 cases (30%) of multiple infections representing 2 genotypes, 12 cases (9%) for 3 genotypes, 5 cases (4.0%) for 4 genotypes and 1 case (1%) for 5 genotypes.

### Potential risk factors associated with HPV infection

The analysis of HPV infection frequency according to women’s sexual behaviour revealed that women with two or more sexual partners were all HPV-positive (Table 3). Those who had their first sexual relationship after the age of 24 years were the most infected with HPV (57.1%). Considering age, women under 30 years were the most infected with HPV (58.4%) compared to other age groups. Besides, all the 3 women who were positive to VIA/VIL were also infected by HR-HPV. However, none of the differences observed in HPV carriage were statistically significant.

**Table 3:** Study of factors associated with HR-HPV infection

	HPV+ (***n*** = 128)	HPV- (***n*** = 112)	Total (***n*** = 240)	***P***-value
**Age n (%)**				0.42
≤ 30	52(58.4)	37(41.6)	89	
31–49	51(49.0)	53(51.0)	104	
≥ 50	25(53.2)	22(46.8)	47	
Total	128(53.3)	112(46.7)	240	
**Age of First Intercourse n(%)**				0.85
0–18	35(50.7)	34(49.3)	69	
18–24	84(54.2.)	71(45.8)	155	
24–30	8(57.1)	6(42.9)	14	
Total	127(53.4)	111(46.6)	238^a^	
**Number of sexual partner n(%)**				0.58
No Partner	54(56.8)	41(43.2)	95	
One Partner	21(50.0)	21(50.0)	42	
Multiple Partners	50(50.0)	50(50.0)	100	
Total	125(52.7)	112(47.3)	237^a^	
**VIA/VIL n (%)**				0.25
Negative	125(52.7)	112(47.3)	237	
Positive	3(100.0)	(0.0)	3	
Total	128(53.3)	112(46.7)	240	

^a^ Results for « age at first intercourse » and « Number of sexual partners » were available respectively for *n* = 238 and *n* = 237 women

## Discussion

The current study on the distribution of high-risk HPV genotypes in the general population of women was conducted in Lomé (Togo). This kind of study on HPV in this city is, to the best of our knowledge, the first one. The objective of this study was to have a precise estimate of the prevalence of high-risk HPV among women in Lomé and identify the most common genotypes in the city in order to create a representative regional map.

### Socio-demographic, sexual, and behavioural characterisation of the women

In this study, the reported age was between 17 and 61 years with an average of 34.67 ± 1.2 years. This population is similar to the study population of Traore et al. [8] in Bobo-Dioulasso (Burkina Faso) whose age varied between 20 and 56 years with an average of 35.3 ± 0.6 years. Married women represented 48.8% of cases, which is less than the findings of Ouedraogo et al. [9] that reported 76% of married women always from the same country (Burkina Faso).

We found a percentage of 52.2% of women which do not use condoms. This can be explained by the fact that most of the women in this study had a married life and therefore used other methods of contraception. The level of knowledge about cervical cancer was low. This could be explained by the relatively low level of education amongst these women and by the lack of information concerning cervical cancer and its high-risk factors.

### Frequencies of precancerous lesions and HR-HPV infection

The prevalence of cervical dysplasia screened by VIA/VIL was 1.3% (3/240). This prevalence is lower than that obtained in Bobo-Dioulasso in Burkina Faso (3.9%) [8].

We found a percentage of 53.3% of women infected with at least one high-risk HPV genotype. This result is greater than that obtained by Zohoncon et al. in 2013 [7] who found a prevalence of 30.2% and that of Ouedraogo et al. in 2015 [10] who found a prevalence of 41.5% in adolescents. It is worth noting that the team of Zohoncon used an HPV detection kit with 12 genotypes. Indeed, previous data about the epidemiology of HPV were obtained from a group of sex workers were the rate of HPV infection was estimated at 66.1% [11]. This was a particularly high-risk group, because some studies have shown that the probability of HPV infection is higher in women with multiple sexual partners [12]. The women in this study reported having only one sexual partner in the majority of cases (81.7%).

The high prevalence of HPV in the current study can be explained by three main reasons: First, the type of population recruited (most of them had genital infections). Secondly, the relatively low level of education of these women and the lack of information concerning cervical cancer and its high-risk factors. Thirdly, the low rate of screening of cervical cancer in Togo since it has been demonstrated that the participation in screening for cervical cancer is associated with a low infection rate of HPV [13].

Furthermore, Singh et al. in 2012 [14], demonstrated that in sub-Saharan Africa, the high prevalence of HPV was due to: immune deficiency, poverty, urbanization, poor socio-economic conditions; the precocity of sexual relationships; numerous maternities without strict hygiene rules.

Some authors reported a link between the number of sexual partners and HPV infection [15]. In the current study, the carrying of the high-risk HPV was not associated with this same risk factor as well others such as age, age at first sexual intercourse, and the result of VIA/VIL. The limit size of our study population could explain this result.

### Distribution of HR-HPV genotypes among the women

The most frequently encountered genotypes were HPV 56, followed by HPV 51 and 31. HPV 16 and 18, targets of the currently available vaccines, were found respectively at 2.3 and 14.01% of cases.

However, our results are similar to those found in several other studies, particularly in West Africa. Indeed, in the study of Traore et al. in Bobo-Dioulasso, Burkina Faso [8], the most frequent genotypes were: HPV 39 (18.51%), HPV 52 (16.66%), HPV 18 (14.81%) and HPV 35 (12.96%). In the study of Djigma et al. in 2011 in Ouagadougou [16], the HPV genotypes found in HIV positive women were HPV50’S (25.5%); HPV30′S (20.8%); HPV16 (4.7%); HPV45 (3.7%). Piras et al. in 2011 in Benin [17] found the following HPV genotypes: HPV59 (24.6%), HPV35 (22.5%), HPV16 (17.6%), HPV18 (14.8%), HPV58 (13.4%), HPV45 (9.9%) and HPV56 (8.4%)**.** In Thailand, the genotypes found were HPV 72 and 52 [18] while in Fiji, the HPV 52, 56, 59 were among the first four high-risk genotypes [19]. All these results indicated a higher frequency of other HR-HPV than HPV 16 and 18.

However, our results are different from the literature that suggests that HPV genotypes 16 and 18 are the most frequent in the world. Indeed, several authors noticed that, in women with a normal cytology and women with an abnormal cytology [20] or women with cervical cancer [21], HPV 16 was the most frequent genotype followed by HPV 18. Similarly, studies on the prevalence of HR-HPV in tumours in France [22] and in Mozambique [23] noted a clear predominance of HPV 16 and 18. The two vaccines, Cervarix (bivalent vaccine HPV 16/18) and Gardasil4 (quadrivalent vaccine HPV 6/11/16/18) that target the HR-HPV 16/18 are available in Togo. Their use will help to effectively protect against genotypes 16 and 18 and thus prevent associated cancers. In our work, we found HPV 56, 51, 31, 52 and 35 among the most frequent high-risk genotypes. The new vaccine, Gardasil9 (HPV 6/11/16/18/31/33/45/52/58), a nonavalent vaccine including HPV 31 and HPV 52 (found ahead of all other genotypes in multiple studies), would be welcome in increasing the prevention of HPV infection in the context of Togo.

## Conclusion

This work allowed us to collect data on the epidemiology and distribution of high-risk HPV genotypes in Lomé. The most frequent genotypes were HPV 56 (22.7%) and HPV 51 (20.3%), followed by HPV 31 (19.5%), HPV 52 (18.8%), and HPV 35 (172%). The five first most common genotypes belong to HPV 30′S and HPV 50′S families. The prognosis of cervical cancer in developing countries is often bad due to its late diagnosis and limited therapy options.

It would be necessary to focus on preventive actions such as information, education and sensitization targeting the population of these countries on HPV infection. Screening of pre-cancerous cervical lesions must be performed on all women while research for new polyvalent vaccines targeting the most frequent genotypes in Africa must continue.

## Supplementary Information

**Additional file 1.**

## Data Availability

The datasets generated and/or analysed during the current study are not publicly available because the study is part of an ongoing project but are available from the corresponding author on reasonable request.

## Abbreviations

CERBA: Pietro annigoni biomolecular research centre
LABIOGENE: Molecular biology and genetics laboratory
HPV: Human papillomavirus
HR-HPV: High risk human papillomavirus
VIA: Visual inspection of the cervix with acetic acid
VIL: Visual inspection of the cervix with lugol
PCR: Polymerase chain reaction
RT-PCR: Real-time polymerase chain reaction
STD: Sexually transmitted disease
WHO: World health Organization
CNE: Ethics committee for research in health of Togo
CERS: Ethics committee for research in health of Burkina Faso
SCJ: SquamoColumnar junction
