# Supplementary material for: Molecular characterization of high-risk human papillomavirus (HR-HPV) in women in Lomé, Togo
Source: BMC Infect Dis. 2021 Mar 19;21:278. doi: 10.1186/s12879-021-05956-5 (PMC7977574; doi:10.1186/s12879-021-05956-5)
Supplement: Supplementary file 1 — Additional file 1. [file 12879_2021_5956_MOESM1_ESM.pdf]

Individual data collection form for the study « Molecular characterization of high-risk human Papillomavirus in women in Lomé (TOGO) »

Date / / / /

1- Surname and First name (s) ……………………………………………… ....

2- Age : …………

3-Occupation: Housewife /_/ Student /_/ Salaried /_/ Retired /_/ Unemployed /_/

4- Address: Phone …………… ……… District ……………… ...

5- Education : Illiterate /_/ Primary /_/ Secondary /_/ University /_/

6- Marital status: Single /_/ Married /_/ Divorced /_/ Widow /_/

7- When did you have your last period?: / / / /

8- At what age did you have your first sexual intercourse? ……. (year)

9- Number of pregnancies :…... Living children……; Miscarriage ……

10- Number of sexual partners:……

11- Do you use a condom during sex? Each time/_/ Never /_/ Sometimes /_/ Rarely /_/

12- Do you use oral contraceptives? Yes /_/ No /_/

13- Frequency of intercourse : 1 per week /_/ 1 per month /_/

14- How many times do you go for a gynecological visit per year ?..........

15- What is the reason for your current visit?

………………………………………………………………………………………

16- Have you ever been screened for cervical cancer ? Yes /_/ No /_/ If yes, how many times ? : ……..

17- What do you think is the cause of cervical cancer ? ………………..

18- Have you ever had a vaginal swab ? Yes /_/ No /_/

19- Have you ever had surgery on your cervix or uterus. Yes /_/ No /_/ If yes, specify the type……………………………………….

20- Have you ever had a gynecological infection ? Yes /_/ No /_/

What kind of infection ? …………………………………………

Thank you for your participation!

This page is for medical staff only

1-Visual screening results

- Result one minute after application of acetic acid (VIA)

  Negatif /_/ Positif /_/

- Result after application of lugol solution (VIL)

  Negatif /_/ Positif /_/

2-Based on the result of the VIA / VIL, what did you suggest to the participant?

Circle the number corresponding to the proposed solution

a. Follow-up advised every ………. Years (fill in the dotted lines with the number of years)
b. Referral to immediate treatment
c. Referral to colposcopy and future treatment
d. Others

   (Specify):……………………………………………………………………………………

3- HR-HPV real-time PCR result

Negatif /_/ Positif /_/

HR- HPV Genotypes	

Date :……/……… /..........
